# Patterns of Sexual Risk Behaviors and Sexuality-Related Risk Factors among Young Adults in Germany: Implications for Prevention and Therapy

**DOI:** 10.1007/s10508-024-02877-7

**Published:** 2024-05-30

**Authors:** Dennis Jepsen, Karl Vince Healy, Marie Bernard, Jenny Markert, Petra J. Brzank

**Affiliations:** 1 Institute of Medical Sociology, Interdisciplinary Center of Health Sciences, Martin Luther University Halle-Wittenberg, Magdeburgerstraße 8, 06112 Halle (Saale), Germany; 2 Institue of Social Medicine, Rehabilitation Sciences and Healthcare Research, University of Applied Sciences Nordhausen, Nordhausen, Germany

**Keywords:** Chemsex, Hypersexual behavior, Intimate partner violence, Sexually transmitted infections, Sex work, Sexual dysfunction

## Abstract

Sexual risk behavior (SRB) includes behavioral (sex without contraception, sexualized substance use, sex work, sexual partner violence, other sexual activities that harm oneself or others) and affective subtypes (sexuality-related feelings of shame/guilt, relationship impairments) and leads to psychosocial and health-related consequences. Young adults comprise a vulnerable group regarding the development of SRB. The study aimed to identify SRB patterns among young adults and their relation to sexuality-related risk factors. A cross-sectional online survey measured behavioral and affective aspects of SRB with nine items. Latent class analysis was conducted to identify patterns of SRB. Gender, sexual orientation, age of first intercourse, number of sexual partners, hypersexuality, and sexual dysfunction were captured as risk factors via multinomial logistic regression. Within this convenience sample (n = 609; n_female_ = 365; n_male_ = 245; M_age_ = 23.1 years), the SRB patterns unremarkable (67%; low values in all SRB subtypes), shame-ridden (17%; high values in sexual feelings of shame/guilt) and risky sexual behavior (16%; high values in all subtypes of SRB, especially sexualized drug use) were identified. The shame-ridden and risky patterns were strongly associated with higher hypersexuality values, the risky pattern moreover with being non-heterosexual, of younger age at first sexual experience, and a higher number of sexual partners. Male and sexual minority participants demonstrated SRB more often than females and heterosexuals. Within prevention and treatment of SRB, it seems beneficial to address sexuality-related feelings of shame/guilt and addictive patterns (concerning sexual behaviors/substances) via gender- and diversity-sensitive measurements.

## Introduction

### Theoretical Framework

Sexual risk behavior (SRB) comprises all sexual behaviors and activities that have social and/or health-related consequences (Hammelstein, 2006). SRB includes sex without contraception, sex under the influence of illegal drugs, sex work (Büttner, 2018; Senn et al., 2008) and sexual partner violence (Jewkes et al., 2010).

Young adults are a considered vulnerable group for engaging in SRB. The life phase of young adulthood is characterized by transitions in several life domains, like work and education, finances and intimate relationships (Murray et al., 2020), before making respective enduring decisions in later developmental phases (Arnett et al., 2014). If these transitions are related to crises or instability they can—depending on available social resources (Lane, 2015)—result in psychological distress and negative health outcomes (Lane et al., 2017; Matud et al., 2020). The implementation of risk behavior, such as SRB, can be considered as a maladaptive coping strategy to compensate these stresses at a young age (Raithel, 2011). Additionally, negative experiences in childhood can influence the sexual behavior of individuals which becomes noticeable with the onset of sexual activity with sexual partners in adolescence and young adulthood. In connection with this, abuse in childhood (especially emotional abuse), unfulfilled attachment needs and related difficulties in impulse regulation (often in combination with substance use) are recognized as possible causes of SRB development (Thompson et al., 2017).

**Fig. 1:**
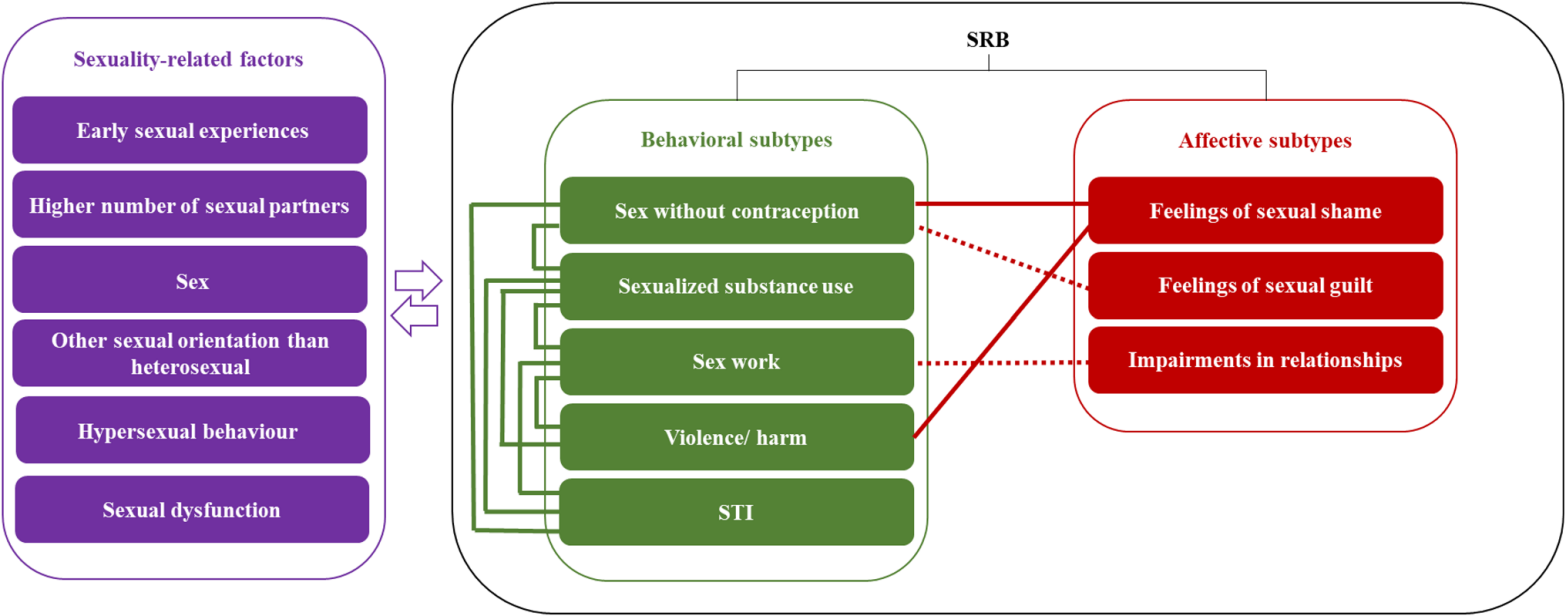
Previous findings on sexual risk behavior and relations between its subtypes and further sexuality-related risk factors, Note: SRB = sexual risk behavior. STI = sexually transmitted infection. Continuous lines stand for risk factors identified in previous research. Dotted lines stand for relations that are not investigated sufficiently

SRB has been investigated thoroughly from different perspectives, using various definitions and observed sexual behaviors. During the review of relevant literature, it became clear that despite behavioral expressions of sexuality, related affective factors seem to be associated with SRB. Figure 1 shows a framework model that aims to overview the current state of research concerning SRB and relations between its single SRB subtypes of behavioral and affective subtypes, as well as associations to further sexuality-related factors that were addressed in the considered studies. In the following, these insights will be described in more detail. As psychosocial and medical consequences of the respective sexual behavior comprise a core feature of the SRB definition and they highlight the relevance of SRB for medical and psychosocial care, they are also displayed in the following.

### The Behavioral Subtypes of Sexual Risk Behavior

#### Sex without Contraception and Sexually Transmitted Infections

Hardly any study could be found that investigated the prevalence of sexually transmitted infections (STIs) among German young adults in particular. However, current findings reveal that only around 60% of German single male young adults use condoms during sexual intercourse (Hintzpeter et al., 2022) and only around 50% of adults used a condom during the last risky sexual contact (Schmidt et al., 2018). At the same time, young German adults seem to be well-educated and better informed about STI than adults above the age of 35 (Matthiesen et al., 2021). However, a prevalence of STI of around 10% was identified among a convenient sample of young adults in Germany (Starostzik, 2021). All these findings suggest considering various target groups differentiated by sociodemographic information, lifeworld, psychosocial aspects and health status, to rethink prevention measures (Matthiesen et al., 2021; Schmidt et al., 2018).

#### Sex Under the Influence of Illegal Drugs

If the consumption of alcohol and other drugs is targeted to evoke specific physical and/or psychological effects during sexual activity (e.g., provide more intense sexual arousal and experience or improved sexual performance), we talk about sexualized substance use (Deimel et al., 2016), which is considered as another behavioral subtype of SRB. Current research concentrates primarily on chemsex, which refers to the use of chemical drugs, like methamphetamine, mephedrone, or gamma hydroxybutyrate (Gertzen et al., 2022), as a form of sexualized substance consumption. Besides the use of illegal drugs, an in general more frequent alcohol use was found to be a risk factor for SRB (Tucker et al., 2022). The interplay between sexuality and substance consumption is assumed to be a destructive circuit that maintains SRB and its psychosocial and medical consequences (Resch & Parzer, 2022). Moreover, substance use was found to be related to both sex without contraception and to STI infections among young adults (Berry & Johnson, 2018; Dessunti & Advincula Reis, 2007; Rosenberger et al., 2021).

#### Sex Work

Sex work represents another subtype of SRB, as sex workers are at a high risk of various hazards to their health and bodily integrity. They are more likely to become victims of physical and sexual violence by intimate partners, clients, or the police (Deering et al., 2014; Johnson et al., 2023; Lutnick et al., 2015) and more likely to be exposed to STIs, esp. HIV (Baral et al., 2012; Burnette et al., 2008). Sex workers are also more frequently diagnosed with substance use disorders than the general population (Burnette et al., 2008). Furthermore, the use of heroin and cocaine at a young age was found to be a predictor for engaging in sex work later in life (Ishøy et al., 2005). It should be noted that sex work is not inherently defined as SRB, though some of its domains and sex workers’ living and working conditions can increase the risk of the mentioned health-related and psychosocial concerns.

#### Sexual Partner Violence

Violent behavior against sexual or intimate partners does not only occur within sex work context. A special form of violent behavior and a further aspect of SRB—which is especially relevant for the target group of this research—is sexual or intimate partner violence among adolescents (also called teen dating violence). About two-thirds of German teenagers report at least one experience of teen dating violence, whereby emotional violence (including verbal aggression, coercion and threats) seems to be the most common type of violence and sexual abuse seems to be the least common (Brzank et al., 2014). These findings are supported by further research with other samples (Taussig & Garrido, 2017). Chemsex practice (Bohn et al., 2020) was identified as a predictor for violent behavior in general and intimate partner violence perpetration (Espelage et al., 2018; Tussey et al., 2021) and victimization (Parker & Bradshaw, 2015) among adolescents in particular.

### The Affective Subtypes of Sexual Risk Behavior

#### Feelings of Sexual Shame and Guilt

According to the objectification theory by Fredrickson and Roberts (1997), “girls and women are typically acculturated to internalize an observer’s perspective as a primary view of their physical selves,” with habitual body monitoring and resulting shame and anxiety regarding their body and sexuality. In particular, women who are ashamed of their bodies (body dissatisfaction), fear negative evaluation and objectify themselves, might not have the confidence to advocate for themselves in terms of acting in accordance with their sexual desires, needs and health (Schooler et al., 2005).

The sociocultural framework theory by Dittmar (2005) postulates that perceived social pressures exerted by significant others (e.g., family members, or the peer group) and mass media cause these concerns and dissatisfaction. In other words, female body satisfaction is and always has been strongly impacted by patriarchal and capitalistic influences (Gopal & Gopal, 2010). Thus, it is not surprising that women are more often affected by body dissatisfaction and its consequences than men (Algars et al., 2009; Quittkat et al., 2019; Tiggemann, 2004). Especially young adult women report body dissatisfaction more frequently than their male counterparts, although the importance of physical appearance seems to decrease with older age (Tiggemann, 2004). Women who are ashamed of their sexuality, body functioning and body image tend to either engage in sexual activity less often or overcompensate with sexual risk-taking. Similarly, women who indicate no feelings of shame regarding their menstruation also report more body satisfaction and less sexual risk-taking (Schooler et al., 2005). Moreover, women who experienced sexism and objectification tend to have more sexual partners and to misuse alcohol and drugs more often than women who have never faced sexism (Ertl et al., 2022).

Not only women are at greater risk of experiencing sex-related shame. For instance, Park et al. (2014) investigated the role of shame among young men who have sex with men (MSM). They found shame for sexual desire to be negatively correlated with knowledge and self-efficacy regarding safer sex among these young men. Moreover, shame was found to be positively associated with risky sexual decision-making, i.e., unprotected intercourse. Further, body shame in men, regardless of their sexual orientation, is considered a predictor of sexually aggressive behavior, especially when masculinity is perceived as threatened (Mescher & Rudman, 2014).

#### Impairments in Relationships

Except for the mentioned studies that could identify relations between sexual/intimate partner violence and other facets of SRB, research on associations between other kinds of impairments in important relationships or individual social networks is scarce. Nevertheless, some relations were found between impairments in important relationships and further sexuality-related risk factors associated with SRB (which are discussed below), like hypersexual behavior (Jepsen & Brzank, 2022) and sexual dysfunction (Basson & Gilks, 2018; Chung et al., 2015).

### Further Sexuality-Related Risk Factors

#### Gender and Sexual Orientation

Previous research has shown that sexual and gender minorities, like lesbian, gay, bisexual, transgender, intersex, queer and other sexual or gender diverse people (LGBTIQ+) and especially MSM (Maxwell et al., 2019), are a particularly vulnerable group for several subtypes of SRB, especially STI (Rasberry et al., 2018). The minority stress theory proposed by Meyer (2003) can be assumed as a potential explanation model for these relations. According to this theory, people whose sexual orientation and gender identity differs from the heteronormative norm (which is heterosexual and cis-gender) face various psychosocial stressors due to their minority status and their related experiences of prejudice and stigma (Hendricks & Testa, 2012). Drawing from this theory, Hatzenbuehler (2009) developed the psychological mediation framework, which suggests that these stresses of sexual minorities lead to cognitive, affective and social changes in psychological processes, which in turn lead to negative mental and sexual health outcomes (Schwartz et al., 2016). Moreover, studies provide evidence that young adult women are more likely to become victims of teen dating violence than young adult men (Puzzanchera, 2022). Women also engage in sex work more often than men. Thus, it is presumed that significant gender- and sexual orientation-related differences exist in SRB and its health-related consequences.

#### Sexual Experiences

Sexual experiences at a young age, often defined as before the age of 13 or 14 (Kaplan et al., 2013), seem to be related to different subtypes of SRB, including alcohol or drug use before sex, not using a condom (Parkes et al., 2014) and being exposed to forced sex and physical violence in dating (Kaplan et al., 2013). Besides early sexual activity, the number of sexual partners in a lifetime or at a particular stage of life may also be associated with SRB. A higher number of sexual partners increases the risk of STI infections. Although sex with protection helps to reduce this risk, a study with longitudinal data shows that people who have had many sexual partners in the previous three months are more likely to have sex without protection (Ashenhurst et al., 2017).

#### Hypersexual Behavior

Clinically apparent hypersexual behavior is classified as compulsive sexual behavior disorder (CSBD; 6C72) in the ICD-11. It is characterized by a persistent pattern of failure to control sexual impulses or urges, resulting in repetitive sexual behavior and impairments in important areas of life, such as family or work life (Bründl & Fuss, 2021; World Health Organization, 2024). Previous research shows that hypersexual behavior seems to be related to several subtypes of SRB, especially to sex under the influence of illegal drugs and to sexual activities that are prohibited by law and are physically or mentally harmful (Jepsen & Brzank, 2022). This is in line with research showing that SRB is a common symptomatic expression of pathologic hypersexual behavior (Briken et al., 2022; Öberg et al., 2017; Rooney et al., 2018). Moreover, hypersexual behavior was identified to be associated with violence victimization in sexual and non-sexual contexts (Chatzittofis et al., 2017; Marshall, 2023).

#### Sexual Dysfunction

The symptoms of sexual dysfunction show gender-related differences. The first study using the new ICD-11 guidelines to estimate the prevalence of sexual dysfunctions in Germany was conducted by Briken et al. (2020). It revealed that the most common sexual dysfunctions among young German men (age 18–25) seem to be premature ejaculation (16.3%), hypoactive desire (10.4%) and delayed orgasm (9.3%). The problem most often reported by women is difficulties in achieving orgasm (27.0%), followed by hypoactive sexual desire (19.4%), sexual arousal problems (16.8%) and sexual pain (16.2%).

Research on the interaction between sexual dysfunction and SRB is scarce. There is one study by Akre et al. (2014) that revealed an association between having multiple sexual partners and erectile dysfunction. Other studies describe sexual dysfunctions as frequent long-term consequences of the regular use of various substances (Diehl et al., 2016; Dolatshahi et al., 2016; Grover et al., 2014; Jepsen et al., 2023; Prabhakaran et al., 2018; Yee et al., 2016). The investigated substances are commonly used within the context of sexualized substance use. Moreover, an often replicated risk factor for sexual dysfunction—as well as for SRB—among both men (Anderson et al., 2022) and women (Bornefeld-Ettmann et al., 2018; Gewirtz-Meydan & Lahav, 2020; Weiss et al., 2023) comprise traumatic experiences in childhood and youth. Since there are some indications of a relationship between SRB and sexual dysfunctions and they seem to share risk factors, it seems beneficial to explore these associations more profoundly.

#### Objectives

The current state of research concerning SRB has shown that it is associated with various health-related and psychosocial consequences that disproportionally affect young adults. Moreover, relations between several subtypes of SRB among each other are well documented in previous research. Hence, the complexity of SRB is a potential challenge for professionals in psychotherapy, social pedagogy and medicine. However, studies on combined SRBs (including patterns and relationships between SRB subtypes, sexuality-related factors and other variables) are lacking. Such research would enhance our understanding of SRB complexity and help identify multifactorial risks and consequences.

In this study, latent class analysis (LCA) was used to identify SRB patterns, since this method is considered especially useful for identifying (vulnerable) “subgroups of individuals who could benefit from a common intervention based on their shared characteristics” (Weller et al., 2020). In the case of SRB, these interventions could be included in prevention, counseling and therapy contexts to reduce risk behavior and related harm. Another advantage of LCA is that it is considered a person-centered approach, which allows the exploring of relationship patterns among individuals of the sample and therefore identifying homogenous groups and not only linear relations between SRB subtypes and related consequences like within variable-centered approaches (Killian et al., 2019). This can be especially beneficial for defining guidelines in prevention and interdisciplinary care.

Only two studies investigating multivariate relations between SRB subtypes could be found. Morales et al. (2021) investigated condom use behavior and sexualized drug use among Spanish adolescents via latent class analysis. They identified four classes. Three classes included SRBs and one did not. About two-thirds of the sample were assigned to the class with unproblematic sexual behavior. In their study, adolescent women were significantly more likely to be assigned to the unproblematic class and the likelihood of engaging in sexualized drug use increased with age. A different study revealed an association between substance abuse and SRB sex with multiple partners via LCA (Connell et al., 2009). No other studies were found that used structural equation models to explore subgroups of individuals affected by SRB. However, employing such approaches can deepen our understanding of SRB by identifying pathways and behavioral structures of sexual risk-taking. This, in turn, forms the foundation for well-conceived prevention and support strategies. Hence, this study aims to explore the complexity of SRB further by including all addressed SRB subtypes of previous research and by identifying their risk factors. This study therefore strives to achieve the following objectives:

1. Identifying relations between all single subtypes of SRB and their potential risk factors.
2. Determining SRB patterns, including their behavioral and affective subtypes, as well as their relation to the identified risk factors.
3. Exploring the relation between SRB and sexual dysfunctions.

Considering the current state of research, it can be anticipated that in our analyses (1) substance use will be related to all investigated subtypes of SRB, (2) sex without contraception and STI will be associated with feelings of sexual shame and guilt, (3) sexuality-related factors like earlier age of first intercourse, sex with multiple partners, gender, sexual orientation and hypersexual behavior will be related to SRB patterns.

## Method

### Participants and Procedure

The target group of this cross-sectional study were young adults in Germany aged 18–27. Data were collected in 2021 using an online survey on the platform SoSciSurvey. The survey was promoted through an online forum addressing sexual topics and through a dating website for casual sex*,* as well as via Facebook and the mail distribution of the University of Applied Sciences Nordhausen (Germany).

### Measures

All items were phrased in gender-equitable and sensitive German language (Muschalik et al., 2021). Nine self-developed items were used to measure subtypes of SRB as dependent variables:

1. Illegal sexual acts: “I have performed sexual acts that are not legally allowed.”
2. Sex work: “I prostituted myself.”
3. Caused physical or emotional harm: “I have performed sexual acts that harmed someone else physically or emotionally.”
4. Sexualized substance use: “I do have sex under the influence of illegal drugs.”
5. Feelings of shame: “I feel ashamed of my sexual activities.”
6. Feelings of guilt: “I feel guilty after sex.”
7. Impaired relationships: “My sexual activities strongly impair important relationships with other people in my life.”
8. Contraception use: “I believe that I use contraception appropriately for my sexual behavior.”
9. STI: “I have already contracted a STD because I did not use adequate contraception during sex.”

The items 1 to 8 measured SRB with five-point Likert scales (response options of items 1 to 7: “never” to “very often.” Response options of item 8: “strongly disagree” to “strongly agree”), item 9 with a nominal scale (response options: “yes,” “no” and “I don’t know). Different perspectives on violent behavior toward sexual partners were measured with the items “I have performed sexual acts that are not legally allowed” and “I have performed sexual acts that harmed someone else physically or emotionally.”

The sociodemographic background of the participants was captured via the factors of age, migration background, gender identity and sexual orientation. Gender identity was measured based on a non-binary understanding. Response options included “female,” “male,” “non-binary,” “other” (with the opportunity of concretization via free text function) and “prefer not to say.” Migration background was identified via the item “Me or at least one of my parents was not born with German citizenship (yes/no).”

Furthermore, the participants were asked about aspects of their sexual life, such as the age of their first intercourse, the total estimated number of sexual partners, hypersexual behavior, and sexual dysfunction. Hypersexual behavior was assessed by the Hypersexual Behavior Inventory (Reid et al., 2011), which measures the construct via 19 items on a five-point Likert scale with response options ranging from never to very often. Participants were classified as hypersexual if they reached the cutoff value of ≥ 53 (Reid et al., 2011). Possible symptoms of sexual dysfunction (premature orgasm, delayed orgasm, failure to orgasm, lack of libido, arousal problems and pain during sex) were captured via seven-point Likert-scale items with response options between “never” and “always.”

### Statistical Analysis

The univariate and bivariate analyses were conducted with IBM SPSS Statistics, version* 27*, the LCA and the multinomial logistic regression with *STATA 15*. Bivariate analyses were conducted via correlation calculations. Mean value comparisons were calculated via chi-square test and Mann–Whitney U test.

The statistical method of LCA (Naldi & Cazzaniga, 2020; Nylund et al., 2007; Oberski, 2016) was used to identify classes with similar sexual (risk) behaviors. LCA uses manifest response variables to detect an underlying (latent) construct with discrete expressions. All LCA-related computations were implemented with the Penn State University LCA Stata plug-in (Lanza et al., 2018) and *STATA 15*. As manifest response variables all items measuring SRB were used. For these variables to serve as a basis for the LCA, they had to be dichotomized. The responses “occasionally,” “sometimes,” “often” and “very often” were collapsed into one response category (value = 1) and the response “never” into the other (value = 0). Thus, the categories “never engaged in SRB” and “at least occasionally engaged in SRB” were compared. Different LCA models with two to seven classes were calculated to identify the optimal class number. Entropy *R*^*2*^ and four different likelihood-based goodness-of-fit statistics served to compare models and select the best one. The likelihood-based fit statistics used to evaluate the different LCA models include the Akaike information criterion (AIC), the consistent Akaike information criterion (CAIC), the Bayes information criterion (BIC) and the sample-size-adjusted BIC (SABIC). A detailed description of these fit statistics can be found elsewhere (Nylund et al., 2007; Weller et al., 2020).

Due to theoretical considerations, we decided against the inclusion of covariates within a three-step LCA. In doing so, we could set the focus on identifying combined risky sexual behaviors within an explorative approach, in line with the objectives of this study. Also, the sexuality-related factors we measured were not covered by the definitions of behavioral and affective patterns of SRB according to relevant literature, which are key points of the theoretical model we oriented on for our analyses. An exception can be hypersexual behavior, which is indeed a behavioral factor, but does not necessarily express as SRB. The analysis of the relations between the identified patterns was necessary for the discussion of our results and the derivation of indications for psychosocial care, but not required for answering the research questions.

Multinomial logistic regression was used to identify the influence of different variables concerning personal and sexual background on latent class membership. In the first step, bivariate models were calculated for each respective independent variable. In the second step, a comprehensive model was calculated including all independent variables. The following variables were used to predict latent class membership:

1. Gender identity (male/female). Since only six participants stated to be non-binary and two participants chose the answer option “other,” they were not included in the data analyses.
2. Sexual orientation (heterosexual/sexual minority). The response options “asexual,” “bisexual,” “homosexual,” “pansexual,” and “other” were collapsed into the category “sexual minority.”
3. Age at first sexual experience (in years).
4. Total number of sexual partners.
5. Sexual dysfunction (positive if at least one of the following occurs often or more): premature orgasm, failure to orgasm, delayed orgasm, lack of libido, arousal problems, pain during sex.
6. HBI-score.

## Results

### Sample Characteristics

The initial sample size was 609. Overall, 58.5% reported female, 40.2% male gender identity and 1.0% stated they were non-binary. Because of the small number of non-binary participants (*n* = 6) and participants who chose the response option “other” (*n* = 2), they were excluded from all the following calculations, resulting in a final sample size of *n* = 601 participants. In total, 65.2% of the participants reported to be heterosexual, 15.4% bisexual, 3.1% homosexual, and 5.8% to have another sexual orientation. The mean age was *M* = 23.1 years (*SD* = 2.7). Migration background was reported by 20.5% (*n* = 123). The average values of reported sexual experiences are shown in Table 1.

**Table 1:** Average values concerning sexual experiences stratified by sex

	Total (n = 601)		Female (n = 365)		Male (n = 245)		Female vs. Male
	M (SD)		M (SD)		M (SD)		*t*, *p*-value
Age of first intercourse	16.7	(2.50)	16.5	(2.32)	17.1	(2.72)	− 2.51, .012
Number of sexual partners	5.7	(4.97)	4.7	(3.66)	9.7	(9.94)	− 4.43, < .001

### General Aspects of Sexual Risk Behavior

The answer frequency of the items measuring SRB stratified by gender identity and sexual orientation, as well as mean score comparisons (female vs. male; heterosexual vs. sexual minority), is shown in Table 2. Men reported engaging in all investigated SRB more often than women (except engagement in sex work), while significant mean score differences were found between sex and engagement in sex work, a believed sufficient use of contraception, feelings of shame and guilt after sex and caused physical/emotional harm and impairments in important relationships because of the sexual behavior. After Bonferroni-Holm correction, the mean value difference of sexual feelings of guilt loses its significance. Furthermore, sexual minority participants show higher mean scores in all measured subtypes of SRB than heterosexuals, with significant differences regarding the engagement in sex work and sexualized substance use, a perceived sufficient use of contraception, feelings of shame and guilt after sex and caused physical/emotional harm and impairments in important relationships because of the sexual behavior.

**Table 2:** Frequency of sexual risk behavior subtypes stratified by sex and sexual orientation

	Total (n = 601)		Female (n = 365)		Male (n = 245)		Female vs. Male	Heterosexual (n = 397)		Sexual minority (n = 148)		Heterosexual vs. sexual minority
	n (%)		n (%)		n (%)		Comparison of means	n (%)		n (%)		Comparison of means
**Illegal sexual acts**												
Never	432	(71.9)	271	(76.1)	161	(65.7)	U = 28867.50, *p* = .077, adj. *p* = .154 Z = -1.77 M_male_ = 1.32, SD = 0.79 M_female_ = 1.19, SD = 0.56	289	(72.8)	105	(70.9)	U = 20131.50, *p* = .151, adj. *p* = .151 Z = -1.43 M_hetero_ = 1.20, SD = 0.60 M_sexmin_= 1.32, SD = 0.79
At least once	76	(12.6)	40	(11.3)	36	(14.6)		43	(10.8)	23	(15.6)	
Missing answer	93	(15.4)	45	(12.6)	48	(19.6)		65	(16.4)	20	(13.5)	
**Sex work**												
Never	465	(77.4)	294	(82.6)	171	(69.8)	**U = 28735.50, *p* = .005, adj. *p* = .020 **Z = -2.79 M_male_ = 1.17, SD = 0.47 M_female_ = 1.19, SD = 0.56	321	(80.9)	103	(69.6)	**U = 18140.50, *p* < .001 **Z = -5.50 M_hetero_ = 1.07, SD = 0.38 M_sexmin_= 1.29, SD = 0.64
At least once	46	(7.6)	19	(5.3)	27	(11.0)		14	(3.5)	26	(17.6)	
Missing answer	90	(15.0)	43	(12.1)	47	(19.2)		62	(15.6)	19	(12.8)	
**Physical or emotional harm**												
Never	457	(76.0)	292	(82.0)	165	(67.3)	**U = 27762.50, *p* < .001 **Z = -3.78 M_male_ = 1.20, SD = 0.48 M_female_ = 1.08, SD = 0.33	307	(77.3)	108	(73.0)	**U = 19760.00, *p* = .009, adj. *p* = .036 **Z = -2.61 M_hetero_ = 1.10, SD = 0.34 M_sexmin_= 1.21, SD = 0.53
At least once	54	(9.0)	20	(5.6)	34	(13.8)		27	(6.8)	21	(14.2)	
Missing answer	90	(15.0)	44	(12.4)	46	(18.8)		63	(15.9)	19	(12.8)	
**Sexualized substance use**												
Never	380	(63.2)	235	(66.0)	145	(59.2)	U = 29902.50, *p* = .359, adj. *p* = .359 Z = -.91 M_male_ = 1.51, SD = 0.98 M_female_ = 1.37, SD = 0.76	271	(68.3)	78	(52.7)	**U = 16998.00, *p* < .001 **Z = -4.67 M_hetero_ = 1.30, SD = 0.70 M_sexmin_ = 1.69, SD = 1.06
At least once	131	(21.8)	77	(21.5)	54	(22.0)		63	(15.9)	51	(34.5)	
Missing answer	90	(15.0)	44	(12.4)	46	(18.8)		63	(15.9)	19	(12.8)	
**Feelings of shame**												
Never	361	(60.1)	240	(67.4)	121	(49.4)	**U = 26266.50, *p* < .001 **Z = -3.73 M_male_ = 1.56, SD = 0.80 M_female_ = 1.35, SD = 0.73	248	(62.5)	80	(54.1)	**U = 18782.50, *p* = .006, adj. *p* = .030 **Z = -2.72 M_hetero_ = 1.37, SD = 0.71 M_sexmin_ = 1.59, SD = 0.87
At least once	151	(25.2)	73	(20.5)	78	(31.8)		87	(21.9)	49	(33.1)	
Missing answer	89	(14.8)	43	(12.1)	46	(18.8)		62	(15.6)	19	(12.8)	
**Feelings of guilt**												
Never	410	(68.2)	260	(73.0)	150	(61.2)	**U = 28861.50, ***p* = .044, adj. *p* = .132 Z = -2.01 M_male_ = 1.33, SD = 0.63 M_female_ = 1.27, SD = 0.70	276	(69.5)	95	(64.2)	**U = 19556.50, *p* = .023, adj. *p* = .046 **Z = -2.27 M_hetero_ = 1.24, SD = 0.59 M_sexmin_= 1.43, SD = 0.87
At least once	102	(17.0)	53	(14.9)	49	(20.0)		59	(14.9)	34	(22.9)	
Missing answer	89	(14.8)	43	(12.1)	46	(18.8)		62	(15.6)	19	(12.8)	
**Impaired relationships**												
Never	402	(66.9)	265	(74.4)	137	(55.9)	**U = 25776.00, *p* < .001 **Z = -4.47 M_male_ = 1.48, SD = 0.83 M_female_ = 1.21, SD = 0.59	273	(68.8)	92	(62.2)	**U = 19218.50, *p* = .011, adj. *p* = .036 **Z = -2.52 M_hetero_ = 1.27, SD = 0.64 M_sexmin_ = 1.46, SD = 0.86
At least once	108	(18.0)	46	(13.0)	62	(25.3)		61	(15.4)	37	(25.1)	
Missing answer	91	(15.1)	45	(12.6)	46	(18.8)		63	(15.9)	19	(12.8)	
**Perceived sufficient use of contraception**^**1**^												
Agreed	414	(68.9)	262	(73.6)	152	(62.1)	**U = 26200.00, *p* < .001 **Z = -3.66 M_male_ = 3.97, SD = 1.12 M_female_ = 4.29, SD = 1.01	13	(3.3)	8	(3.9)	**U = 17528.00, *p* < .001, adj. *p*** **= .008 **Z = -3.47 M_hetero_ = 4.25, SD = 1.03 M_sexmin_= 3.91, SD = 1.14
Not agreed	101	(16.9)	48	(13.5)	53	(21.6)		326	(82.1)	168	(82.4)	
Missing answer	86	(14.3)	46	(12.9)	40	(16.3)		58	(14.6)	28	(13.7)	
**STI because of inadequate contraception use**												
No	452	(75.2)	274	(77.0)	178	(72.7)	χ^2^(1) = .078, *p* = .780 V = .013, *p* = .780	299	(75.3)	108	(73.0)	χ^2^(1) = 1.315, *p* = .252 V = .054, *p* = .252
Yes	43	(7.2)	27	(7.6)	16	(6.5)		26	(6.5)	14	(9.5)	
I don’t know	25	(4.2)	12	(3.4)	13	(5.3)		16	(4.0)	7	(4.7)	
Missing	81	(13.5)	43	(12.1)	38	(15.5)		56	(14.2)	19	(12.8)	

Note: hetero = heterosexual. sexmin = sexual minority. STI = Sexually transmitted infection. All stated sexual orientations differing from heterosexual are defined as “sexual minority.” ^1^ The answer options “totally agree” and “rather agree” were summarized as “agreed.” n = Absolute frequency. U = Results of Mann–Whitney U test. adj. *p* = adjusted *p*-value after Bonferroni-Holm correction. SD = Standard deviation. χ^2^ = Chi-square test. V = Cramers V. Bold: significant results

Table 3 shows all correlations between the investigated behavioral and affective subtypes of SRB. The strongest correlations were identified between feelings of shame and feelings of guilt after sex (*r* = 0.58, *p* ≤ 0.001), as well as between impairments in important relationships because of the sexual behavior and feelings of shame (*r* = 0.38, *p* ≤ 0.001) and guilt (*r* = 0.31, *p* ≤ 0.001) after sex.

**Table 3:** Correlations between behavioral and affective subtypes of sexual risk behavior

	Illegal sexual acts	Sex work	Physical/emotional harm	Sexualized substance use	Feelings of shame	Feelings of guilt	Impaired relationships	Inappropriate contraception use	STI
Illegal sexual acts	1.00								
Sex work	.20***	1.00							
Physical/emotional harm	.26***	.13**	1.00						
Sexualized substance use	.19***	.20***	.24***	1.00					
Feelings of shame	.15***	.23***	.20***	.16***	1.00				
Feelings of guilt	.05	.17***	.10*	.06	.58***	1.00			
Impaired relationships	.22***	.19***	.19***	.26***	.38***	.31***	1.00		
Inappropriate contraception use	-.11*	-.14**	-.15**	-.13**	-.04	-.10*	-.13**	1.00	
STI	-.18***	-.25***	-.19***	-.16***	-.21***	-.16***	-.26***	.17***	1.00

*** p ≤.001. **  *p* ≤.010. * * p* ≤ .050

The HBI cutoff value was reached by 10.5% of the sample, and hence they were classified as hypersexual. Significant weak to moderate correlations were found between the HBI-score and the following aspects of SRB: illegal sexual activities (*r* = 0.29), offering of sex work (*r* = 0.30), sexual activities that harmed someone emotionally or physically (*r* = 0.32), sex under the influence of illegal drugs (*r* = 0.26). Stronger correlations were identified between the HBI-score and feelings of guilt (*r* = 0.45) and shame (*r* = 0.54) regarding sexual behavior as well as impairments in important relationships as a consequence of the sexual behavior (*r* = 0.61). The HBI-score was further negatively related to the item “I believe that I use contraception sufficiently for my sexual behavior” (*r* = -0.35). No correlation was found between HBI-score and an STI. Weak significant correlations were found between feelings of guilt after sex and the frequency of delayed orgasm (*r* = 0.20) and erectile problems (*r* = 0.25), as well as the HBI-score and the frequency of premature orgasm (*r* = 0.26). There were no other significant correlations between the frequency of sexual dysfunction and the further scales measuring SRB or the HBI-score.

### Patterns of Sexual Risk Behavior via Latent Class Analysis

**Fig. 2:**
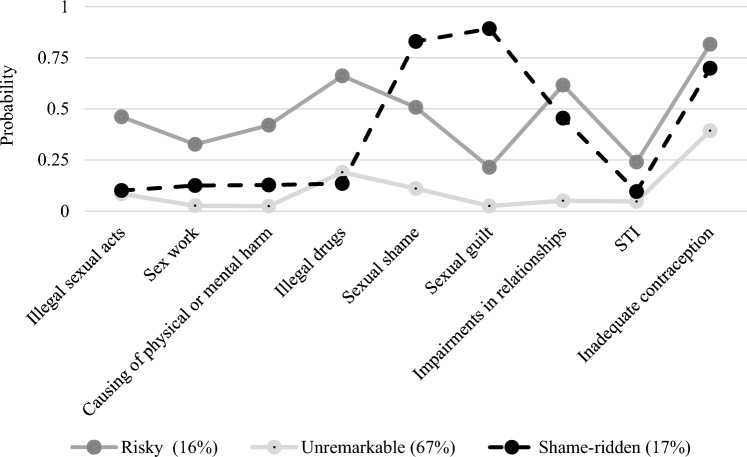
Expressions of sexual risk behavior subtypes within the three latent classes. Note: N = 530. SRB = sexual risk behavior. LCA = latent class analysis. STI = sexually transmitted infection

Six different latent class models were computed, with class numbers ranging from two to seven. Table 5 (Appendix) indicates the goodness-of-fit statistics for each model. The three-class model provides the lowest values for three of the four fit statistics. Because lower values indicate a better fit, the three-class solution was chosen as the best model. Thus, the classes “unremarkable” (67% of the sample), “shame-ridden” (17% of the sample), and “risky” (16% of the sample) were defined. In Fig. 2, the respective emphases of different subtypes of SRB within the three classes are shown.

The likelihood of participants associated with “unremarkable” class showing SRB is low, at least for most subtypes. Thus, approximately every fifth of them is engaged in sexualized substance use at least occasionally and around a third perceive their contraception use as insufficient for their sexual behavior. Over two-thirds of the participants classified as “shame-ridden” reported feelings of shame and guilt regarding their sexual activities. Approximately one-half of shame-ridden participants reported impairments in important relationships as a consequence of their sexual behavior and two-thirds perceived inadequate use of contraception, at least occasionally. Conversely, participants who were classified as SRB type “risky” showed the highest rates for perceived insufficient use of contraception (approx. 80%), followed by sex under the influence of illegal drugs and sexual activities that impair important relationships (respectively, around two-thirds of the members). Further, every second associated participant reported feelings of shame and sexual activities that harmed someone, whereas STI infections and feelings of guilt regarding the sexual behavior at least occasionally were reported by every fifth.

A multinomial logistic regression was conducted to identify the influence of different variables concerning personal and sexual background on latent class membership. The respective results are shown in Table 4. In the bivariate models, men are 4.03 times more likely than women to belong to the class of “risky” sexual behavior and 2.9 times as likely to belong to the “shame-ridden” class than to the class of remarkable sexual behavior. Furthermore, for every additional sex partner, the odds of belonging to the “risky” class increase by 1.17 in comparison with the “unremarkable” class. If respondent A had sex with 5 people, he/she/they is 1.85 times more likely than respondent B who had sex with 0 people to belong to the “risky” class compared to the “unremarkable” class (5*0.17 + 1 = 1,85). Respondent C with 10 sexual partners is 2.7 times more likely than A to belong to the risky class (10*0,17 + 1 = 2,7).

**Table 4:** Results of multinomial regression

Sexual behavior	Risky^a^				Shame-ridden^a^			
	Bivariate models		Complete model		Bivariate models		Complete model	
Variable	OR	95% CI	OR	95% CI	OR	95% CI	OR	95% CI
Gender (reference: Female)	**4.03**	**[2.07;7.86]**	0.78	[0.17;3.61]	**2.90**	**[1.48;5.67]**	0.81	[0.31;2.12]
Sexual orientation (reference: Heterosexual)	**6.96**	**[3.23;15.01]**	**6.19**	**[1.41;27.26]**	1.96	[0.85;4.52]	1.64	[0.65;4.17]
Age at first sexual experience	**0.55**	**[0.43;0.70]**	**0.59**	[0.37;0.93]	1.03	[0.91;1.17]	1.02	[0.85;1.22]
Total number of sexual partners	**1.17**	**[1.11;1.23]**	**1.08**	**[1.01;1.14]**	**1.07**	**[1.00;1.13]**	1.02	[0.97;1.07]
Sexual dysfunction (reference: No dysfunction)	1.13	[0.60;2.14]	0.43	[0.08;2.26]	**2.25**	**[1.10;4.62]**	1.82	[0.73;4.55]
HBI-score	**1.21**	**[1.15;1.28]**	**1.22**	**[1.12;1.32]**	**1.19**	**[1.12;1.25]**	**1.18**	**[1.12;1.25]**

n = 387. OR = odds ratio. CI = confidence interval. HBI = Hypersexual Behavior Inventory. Bold: CI associated with OR does not include 1 (the effect is statistically significant). ^a^Reference class: unremarkable sexual behavior

Sexual dysfunction was statistically non-significant in both complete models. Hypersexuality mediates gender assignment, as men are more prone to hypersexuality and as a result more likely than women to belong to the class of “risky” and the class of “shame-ridden” behavior. However, gender, the number of sex partners, and sexual dysfunction lose their significance from the bivariate models to the complete model concerning the “shame-ridden” class. Hypersexuality remained the only significant variable here. Concerning the “risky” class, sexual minority status, younger age of first sexual experience, higher number of sexual partners, and higher HBI values remained significant predictors of class membership.

## Discussion

The aims of this study were (1) identifying relations between all single subtypes of SRB and their potential risk factors, (2) determining SRB patterns, including their behavioral and affective subtypes, as well as their relation to the identified risk factors, and (3) exploring the relation between SRB and sexual dysfunctions among this sample of young adults. As assumed based on previous research and our hypotheses, sexualized substance use was prevalent in all identified SRB classes, albeit to a varying extent. Also, consistent with the hypotheses, a perceived inadequate use of contraception (but not a STI because of inadequate contraception use) was likely to occur among participants with higher values in sexual feelings of shame and guilt. The sexuality-related factors age of first intercourse, total number of sexual partners, sexual orientation and hypersexual behavior were identified as risk factors SRB patterns relevant from a therapeutic perspective. No association was found between any form of sexual dysfunction and SRB. Derived from the results of this study, indications for prevention and therapy will be discussed in the following:

### Patterns, Risk Factors, and Implications for Prevention and Therapy

There is empirical evidence for the effectiveness of sexual health risk reduction interventions. Educational or behavioral therapeutic interventions, motivational exercises and counseling can foster positive attitudes toward condom use, condom-protected sexual activity and knowledge about STIs (Henderson et al., 2020; Pandor et al., 2015). Interventions that aim to reduce sex-related shame can also reduce SRB, i.e., unsafe sex (Christensen et al., 2013). In particular, some studies indicate an respective effectiveness of motivational interviewing (Bassett et al., 2022; Flickinger et al., 2013) and skills training (Calsyn et al., 2009). However, previous research on SRB, as well as the results of this study emphasize that safer sex is not the only subtype of SRB which should be considered in prevention and treatment and a more complex understanding of SRB is necessary to provide adequate care for help-seekers.

LCA revealed three patterns of SRB with different underlying behavioral and affective subtypes. Two-thirds of the sample can be placed in the “unremarkable” group and in principle, most investigated characteristics seem inconspicuous from a therapeutic perspective. However, the at least occasional consumption of illegal drugs and especially a perceived inadequate contraception use was reported in this group quite often (although the probability of sexualized drug use is lower than in the other classes). Therefore, it seems beneficial to also keep an eye on this SRB pattern when conceptualizing SRB prevention and support interventions. This can be done by recognizing the close interaction between substance use and sexuality, especially since this interaction plays a role in both chronic substance use and sexual disorders (Hallinan, 2021), as well as in other health-related factors like STI (Berry & Johnson, 2018; Dessunti & Advincula Reis, 2007; Rosenberger et al., 2021) or intimate partner violence perpetration and victimization (Espelage et al., 2018; Parker & Bradshaw, 2015; Tussey et al., 2021).

Feelings of shame and guilt regarding sexual activities seem to play an important role concerning SRB among the young adults of this sample. Firstly, significant correlations were found between sexuality-related feelings of shame and guilt and impairments in important relationships because of sexual behavior. On the other hand, at least occasionally occurring feelings of shame and guilt regarding sexual activities, as well as a perceived inadequate use of contraception and impairments in important relationships appear predominantly within the determined “shame-ridden” SRB pattern. Since the probability of other investigated forms of SRB is low within this class, it seems expedient to explore its members’ sexual insecurities within prevention and support interventions and to estimate if the performed sexual behaviors are indeed problematic from a therapeutic perspective. As described above, there are various possible explanations for the development of sexuality-related feelings of shame and guilt, which should be addressed in prevention and therapeutic interventions.

Higher values in hypersexuality increase the risk of belonging to the “shame-ridden” group, and thus another way to possibly improve the treatment of SRB is to investigate whether these feelings of shame/guilt reduce or disappear when hypersexuality symptoms are alleviated or—vice versa—whether symptoms of hypersexuality reduce when shame and guilt are reprocessed during therapy. First studies on this perspective indicate that negative health outcomes of hypersexual behavior can be reduced if feelings of sexual shame and guilt are decreased among men from sexual minorities (Cienfuegos-Szalay et al., 2022).

The probability of engaging in sexualized substance use at least occasionally is around 70% within the “risky” group. Other related factors, especially an at least occasionally occurring inadequate use of contraception and impairments in important relationships, might then comprise a direct consequence of the substance use and its cognitive effects. In this case, it seems beneficial to generate awareness of sexual problems and risk behaviors as common adverse effects of regular substance use (Hallinan, 2021; Resch & Parzer, 2022) within prevention programs. Furthermore, alcohol and drug problems should be addressed in therapy and counseling services that address sexual impairments and vice versa. This should help to explore any associations between substance use and sexual problems and clarify adequate further steps of psychosocial support. Depending on the specific case, it can be necessary for the affected person to first attend addiction therapy or counseling and to explore afterward if the sexual problems can be alleviated with the help of appropriate interventions.

### Value of Gender and Sexual Orientation

Slight mean value differences were found regarding gender identity, indicating that men report higher rates of sexual activities that harmed someone physically or mentally, feelings of guilt regarding their sexual behavior and impairments in important relationships as a consequence of their sexual behavior, at least occasionally. Conversely, women show slightly higher rates of sex work but also tend to be more confident about the sufficient use of contraception. Thus, SRB seems to express itself differently among women and men, which should be considered in the conceptualization of prevention programs and therapy. Moreover, further mean value differences reveal that people who report sexual orientations other than heterosexual show higher average rates in sexual behaviors that harmed someone physically or emotionally, sex under the influence of illegal drugs, feelings of shame regarding sexual behaviors and impairments in important relationships as a consequence of their sexual behavior. Thus, consistent with previous research, this study shows that people who do not state to be heterosexual comprise a vulnerable group for SRB and its related medical and psychosocial consequences (Agwu, 2020; Bowring et al., 2015; Deimel et al., 2016; Romero-Estudillo et al., 2014); probably, as a consequence of the stresses sexual minorities are affected by according to sexual minority theory.

Conversely, it is important to take a gender- and diversity-sensitive perspective on SRB while at the same time exploring the life situation of help-seeking individuals, as well as choosing adequate content and methods for prevention and treatment to cope with stresses related to the sexual orientation and gender identity (Chaudoir et al., 2017; Frost & Meyer, 2023). Results of current relevant research, therefore, indicate to use interventions that reduce internal minority stress, as well as related factors like for example self-hate (Nappa et al., 2022), substance abuse (Dermody et al., 2020; Oginni et al., 2022), or loneliness (Skakoon‐Sparling et al., 2023; Torres & Gore-Felton, 2007), as these factors seem to be associated to SRB among sexual minority individuals. At the same time, it is especially crucial to reduce structural stigma, prejudice and discrimination in the public to face this problem on a macro-level (Schwartz et al., 2016).

### Considering Young Adults as Vulnerable Group

As discussed above, young adulthood is characterized by crucial life transitions that can be related to crises and instability and thus cause distress and insecurities. Additionally, childhood trauma and negative attachment experiences at a younger age can be processed during sexual activities, which becomes relevant, especially with the onset of sexual activity including partners, typical for this life stage. Even though this study cannot provide information if and to what extent the occurrence and expression of SRB among young adults differs from other age groups, various risky sexual behaviors were reported quite frequently in our sample. Thus, this study supports the indications of previous studies that young adults should be considered a vulnerable group for engaging in SRB and are affected by its medical and psychosocial consequences in the short and long term.

Diverse risk factors of SRB development could be identified; hence, it is necessary to compensate them with the help of adequate prevention and treatment interventions as soon as possible. In doing so, the above-mentioned gender- and diversity-sensitive perspective on SRB has to be taken into account, as information about sexuality and reproductive and sexual health is often tainted by belief and gender-based assumptions, as well as moral judgments (Gunning et al., 2020). Studies have shown that particularly women and gender minorities are often socialized with negatively connoted messages about their reproductive and sexual health, which can lead to insecurities and shame, shaping the individual sexual behavior (Rubinsky & Cooke-Jackson, 2017).

But not only SRB seems to be a common sexual problem among young adults, since high frequencies of occurring sexual dysfunctions were reported quite often by the participants. Even though no association was found between prevalent sexual functioning problems and SRB, the results of the univariate analysis indicate a need to consider sexual dysfunction among young adults in prevention and therapy. Further research is necessary to explore possible risk and related factors, as well as further psychosocial and medical consequences, specifically among young adults in comparison with older age groups.

Finally, considering the results of previous research and the relevant literature addressing risk factors of SRB, treatment should be integrative, including approaches of behavioral, psychodynamic and sexual therapy. As hypersexual behavior is related to both SRB classes that indicate psychosexual problems (“shame-ridden” and “risky”), it can be assumed that therapeutic interventions to treat hypersexual behavior could help to reduce SRB and thus also reduce the risk of further related medical and psychosocial consequences. It is necessary to explore how prevalent SRB and hypersexual behavior interact with each other and the individual life situation and biography of the persons concerned, while also considering the often reported associations between hypersexual behavior and early traumatic experiences (Slavin et al., 2020). Therefore, while developing and choosing adequate support and treatment, SRB should be considered with all its facets as well as its behavioral, cognitive and affective components.

### Limitations and Directions for Future Research

While interpreting the presented results, some limitations of the study should be considered. Firstly, the selection of the sample could be biased, as the participants were recruited on a website for addiction self-care and a website for casual dating. Since we did not assess through which website or platform participants have been recruited, we were not able to estimate the extent of the possible selection bias. Additionally, SRB contradicts certain moral concepts in a broad section of society; hence, it is possible that statements regarding SRB could be biased due to impression management or social desirability (Hammelstein, 2006).

There are some additional limitations concerning the HBI: In 2022, approximately one year after data collection, the new diagnosis of CSBD was introduced in ICD-11. Based on this newly introduced classification, an instrument considering the new determination of clinical-relevant hypersexual behavior was adapted by Böthe et al. (2020). Moreover, further studies should investigate different forms of SRB in more detail, since the wording of some of the items we used to measure SRB was not sufficiently specific. Especially the factors of sex under the influence of illegal drugs (e.g., regarding the related substance, consumption pattern, manifestation of related addictions), sexual activities that are legally not allowed (e.g., paraphilic activities, sexual abuse, violence in sexual relationships) and sexual activities that harmed someone else (to find out which person was harmed, such as the intimate partner, family members, or others and how they were harmed) should be investigated with more detailed additional items.

LCA is an adequate method for explorative data analyses (Bacher & Vermunt Jeroen, 2010). However, some general limitations regarding LCA should be considered while interpreting the results of this study (Weller et al., 2020). Class assignment is based on probability; thus, an adequate allocation cannot be guaranteed and the frequency of participants assigned to the classes cannot be determined sufficiently. Besides that, the items for SRB measurement were dichotomized in the groups “never engaged in SRB” and “at least occasionally engaged in SRB.” Thus, the results cannot provide detailed information about the frequency of SRB engagement of the respective participants. Nevertheless, the findings can indicate which SRB experiences are likely to occur simultaneously within the respective classes and assigned participants over time. Even though participants engage at least occasionally in SRB, they are or were exposed to the risks related to it and thus, the relevance of psychosocial support remains.

Moreover, there are additional factors that were found to be associated with SRB in previous research that we did not survey. For example, several studies could identify associations between SRB and traumatic stress-related experiences, especially childhood maltreatment (Bozzini et al., 2021; Wang et al., 2019), childhood sexual abuse (Fergusson et al., 2013; Ménard & MacIntosh, 2021; Senn et al., 2008) and prevalent trauma symptoms (Thompson et al., 2017). Especially, MSM (Boroughs et al., 2015; Lloyd & Operario, 2012; Schafer et al., 2013) and transgender women (Scheer & Antebi-Gruszka, 2019) comprise vulnerable groups for these relations. Attachment anxiety (Kim & Miller, 2020) and temperament origin in terms of sensation seeking, stress control, or poor behavioral control (Parkes et al., 2014) were also found to be linked to SRB. Conclusively, it should be considered that the presented results are based on a data collection of a convenient sample, and thus they cannot be derived to other samples or even to the general population.

Conclusively, information about race/ethnic identity was not measured, which limits the discussion of health disparities of marginalized groups. For historical reasons, there are some problems specific to German-speaking areas regarding the use of the terms race and ethnicity in everyday language, as well as in scientific terminology. These terms are seen as a relic of colonial language and thinking within the German language (Fischer et al., 2019). Although we did collect data about the migration background of the participants, these data do not account for social determinants linked to race or ethnic identity, which may contribute to disparities. It is important to note that individuals without a migration background can also experience racism (Kajikhina et al., 2023). Our study was conducted in 2021, while the official recommendations for the operationalization of respective variables by Kajikhina et al. (2023) were published in 2023. Because of disagreements in the scientific discourse before the release of the official recommendations, we could not find an appropriate way for the operationalization of race/ethnic identity.

### Conclusion

When considering SRB in the contexts of prevention and treatment, it is necessary to understand the complexity and variety of its expression, as well as explore potentially associated factors of individual life situations in a gender- and diversity-sensitive way. This study identified three patterns of SRB and can support this assumption of complexity. As high values in hypersexual behavior measurement seem to be a highly influential risk factor concerning the membership to the two identified therapeutic-relevant classes of SRB, the treatment of hypersexual behavior or compulsive sexual behavior disorder could be a beneficial intervention to reduce SRB among young adults. Further research is needed to examine whether the identified classes can be found outside this study’s sample of young adults.

## Data Availability

The participants were ensured that the information collected would be processed solely within the scope of the research project and not shared with third parties. Thus, the datasets generated and/or analyzed during the current study are not publicly available but are available from the corresponding author upon reasonable request.

## Appendix

See Table 5.

**Table 5:** Goodness-of-fit statistics of Latent Class Analysis

Class number	LL	AIC	BIC	CAIC	SABIC	Entropy R^2^
2	-1845.9499	403.21	484.39	503.93	424.08	0.69
3	-1845.9499	352.59	**476.50**	**505.50**	**384.45**	0.77
4	-1845.9499	347.82	514.47	553.47	390.67	0.78
5	-1845.9499	**347.48**	556.85	605.85	401.31	0.73
6	-1845.9499	354.10	606.20	665.20	418.92	0.75
7	-1958.1357	362.37	657.20	726.20	438.17	0.77

LCA = Latent class analysis. LL = Log-likelihood. Likelihood-based fit statistics: AIC = Akaike information criterion. BIC = Bayesian information criterion. CAIC = consistent Akaike information criterion. SABIC = sample-size-adjusted Bayesian information criterion.Bold indicates the best fit for the respective statistic (lower values indicate better fit for all given likelihood-based fit statistics)
